# Supplementary material for: Investigation of the monopole magneto-chemical potential in spin ices using capacitive torque magnetometry
Source: Nat Commun. 2022 Jul 2;13:3818. doi: 10.1038/s41467-022-31297-1 (PMC9250528; doi:10.1038/s41467-022-31297-1)
Supplement: Supplementary file 1 — Supplementary Information [file 41467_2022_31297_MOESM1_ESM.pdf]

Supplementary Information: Investigation of the monopole magneto-chemical potential in spin ices using capacitive torque magnetometry

Naween Anand[1,†], Kevin Barry[1,2,‡], Jennifer N. Neu[1,2,§], David E. Graf[1], Qing Huang[3], Haidong Zhou[3], Theo Siegrist[1,4], Hitesh J. Changlani[1,2], and Christianne Beekman[1,2,*]

[1]National High Magnetic Field Laboratory, Tallahassee, FL 32310, USA
[2]Florida State University, Department of Physics, Tallahassee, FL 32306, USA
[3]University of Tennessee, Department of Physics, Knoxville, TN 37996, USA
[4]Florida Agricultural and Mechanical University and Florida State University, College of Engineering, Tallahassee, FL 32310, USA
[†]present address: Intel Corp., Portland, OR 97006, USA
[‡]present address: Ateios Systems, Newberry, IN 47449, USA
[§]present address: Oak Ridge National Laboratory, Nuclear Nonproliferation Division, Oak Ridge, TN 37831, USA
[*]corresponding author: beekman@magnet.fsu.edu

1 The (001) rotational plane

Supplementary Figure 1 shows the schematic of the capacitive torque magnetometry (CTM) set-up and a 3D representation of the rotation planes. It also highlights the magnetic phases that we experimentally observe in both rotational planes when the magnetic field is aligned with the various high symmetry axes that lie on these planes. Based on the rotation schematics shown in Supplementary Figure 1(c), the measured magnetic torque component is given as

$$\tau_n = \hat{n} \cdot \tau = [0, 0, \bar{1}] \cdot (\mathbf{m} \times \mathbf{B}) \qquad \text{with} \quad \mathbf{B} = B[\cos\theta, \sin\theta, 0] \tag{1}$$

with B the strength of the applied field. When rotating in the (001) plane, the zero crossings are observed at intervals of 45° and sharp turnovers in the data occur near any of the $\langle 110 \rangle$ family of directions. The system always remains in one of the Pauling states in this plane, in particular (2:2)$_0$ (or $Q = 0$[1]), with the field direction determining which of the 6 equivalent Pauling states dominates. These states are characterized by a saturated moment pointing along either the $\hat{x}$ or $\hat{y}$ or $\hat{z}$ directions. For our chosen plane of rotation, it is either along $\hat{x}$ or $\hat{y}$. We observe a sharp turnover in the data with a zero torque crossover points at or near the $\langle 110 \rangle$ directions. This is when all β-spins flip and $\mathbf{m}$ sharply rotates by 90°. The intermediate phases that occur near the $\langle 110 \rangle$ directions could be understood as an equal volume fraction mixture of (2:2)$_0$ phases with half of the sample having $\mathbf{m} \parallel \hat{x}$ and other half of the sample having $\mathbf{m} \parallel \hat{y}$. This would result into a measured saturation magnetization of about 4.1 μ_B/ Ho^{3+} which is same as for the (2:2)$_X$ phase. However, in this rotation plane, we do not see any evidence of a stable (2:2)$_X$ phase as that would require a long range ordering among the β-spins.

Table 1. Functional forms for calculated torque curves for the magnetic spin textures associated with the (001) rotational plane that are shown as solid/dotted curves in Figure 3 of the main text. Note, the magnetization, $\mathbf{m}$, is given as the magnetization per one unit cell, i.e., for 16 Ho^{3+} sites, with μ=10μ_B per site. Transitions between the indicated spin textures requires reversal of 4 β-spins, as indicated in red.

Phase	$\mathbf{B}$-direction	$\mathbf{m} = \sum_{i=1}^{16} \mathbf{m_i}$		τ_n = $[0, 0, \bar{1}] \cdot (\mathbf{m} \times \mathbf{B})$
(2:2)$_0$	$[100]$	$\frac{\mu}{\sqrt{3}}(16, 0, 0)$		$\frac{-16\mu B}{\sqrt{3}} \sin\theta$
50:50 domains	$\langle 110 \rangle$	$\frac{\mu}{\sqrt{3}}(8, 8, 0)$	4xβ	$\frac{\mu B}{\sqrt{3}}(-8\sin\theta + 8\cos\theta)$
(2:2)$_0$	$[010]$	$\frac{\mu}{\sqrt{3}}(0, 16, 0)$	4xβ	$\frac{16\mu B}{\sqrt{3}} \cos\theta$

For each of the mentioned phases the torque response in a given applied field as a function of angle θ, defined as the angle away from the $[100]$ direction, can be calculated using Supplementary Equation 1. Supplementary Table 1 shows the phases,

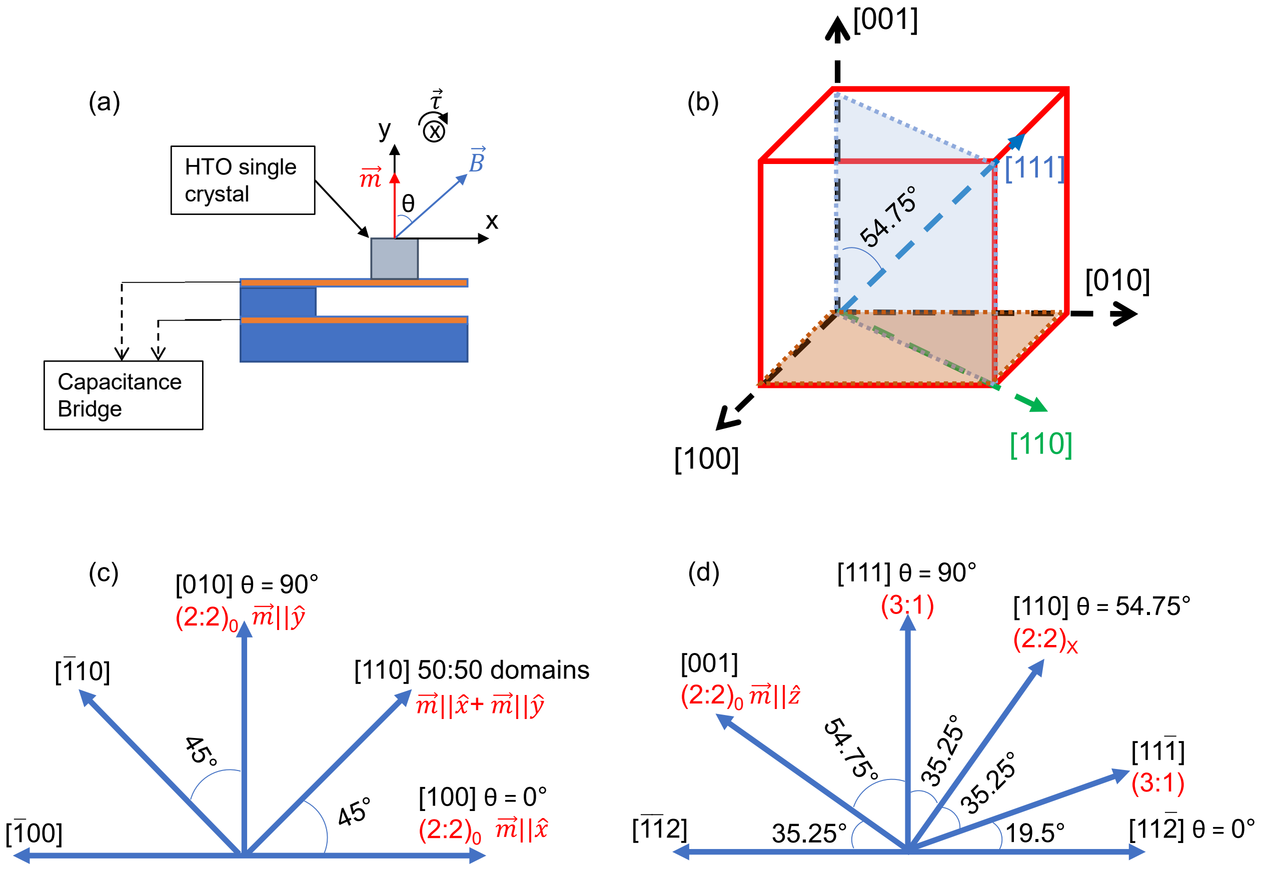

Figure 1. (a) Schematic diagram of the CTM set-up, which shows the sample placement on a flexible BeCu cantilever acting as the top plate of a parallel plate capacitor. A torque (τ) is generated when the magnetic moment (**m**) is not aligned with the applied field (**B**). The angle between **m** and **B** is indicated by θ. (b) 3D representation of the cubic unit cell showing the two color-coded rotational planes of our interest, (001) (tan) and $(1\,\bar{1}\,0)$ (light blue). Rotational schematics for $Ho_2Ti_2O_7$ (HTO) single crystals. (c) (001) plane with magnetic field **B** rotating through the $[1\,0\,0]\rightarrow[1\,1\,0]\rightarrow[0\,1\,0]\rightarrow[\bar{1}\,1\,0]\rightarrow[\bar{1}\,0\,0]$ directions. (d) $(1\,\bar{1}\,0)$ plane with **B** rotating through the $[1\,1\,\bar{2}]\rightarrow[1\,1\,\bar{1}]\rightarrow[1\,1\,0]\rightarrow[1\,1\,1]\rightarrow[0\,0\,1]$ directions. Angular locations for important crystallographic directions and associated magnetic phases are included for both planes.

the associated net magnetic moments, and the functional forms of the torque curves as **B** rotates in the (001) plane. Transitions between spin textures, which can involve spin flips on either the α or β sublattice, lead to gradual changes in the value for $\mathbf{m} = \frac{\mu}{\sqrt{3}} \cdot \mathbf{S}$, with $\mu = 10\mu_B$ the moment per Ho^{3+} ion and with $\mathbf{S} = (S_x, S_y, S_z)$. In the (001) plane, transitions involve only β-spin flips and each flip leads to $\Delta S_x = \pm 2$ and $\Delta S_y = \pm 2$ while S_z remains zero. The torque curves for these intermediate spin textures are calculated in the same way as the other curves, taking into account only this change in **S** (see Figure 3 of the main text for the calculated torque response as a function of $B(\theta)$). The field-angle phase diagram for the (001) rotational plane as determined from the CTM measurements, is provided in Supplementary Figure 2(a).

2 The $(1\,\bar{1}\,0)$ rotational plane

In the plane that contains the body diagonal $[1\,1\,1]$, a similar procedure is used to calculate the angular dependence of the torque response. For this plane, shown in Supplementary Figure 1 (d) and with $B \geq 2$ T, the torque response shows additional zero crossings and turnovers with significant changes in the observed torque amplitude when approaching different high symmetry axes. Note, in this rotational plane α-spins are involved in the phase transition between (2:2)$_0$ and the (3:1) monopole phases

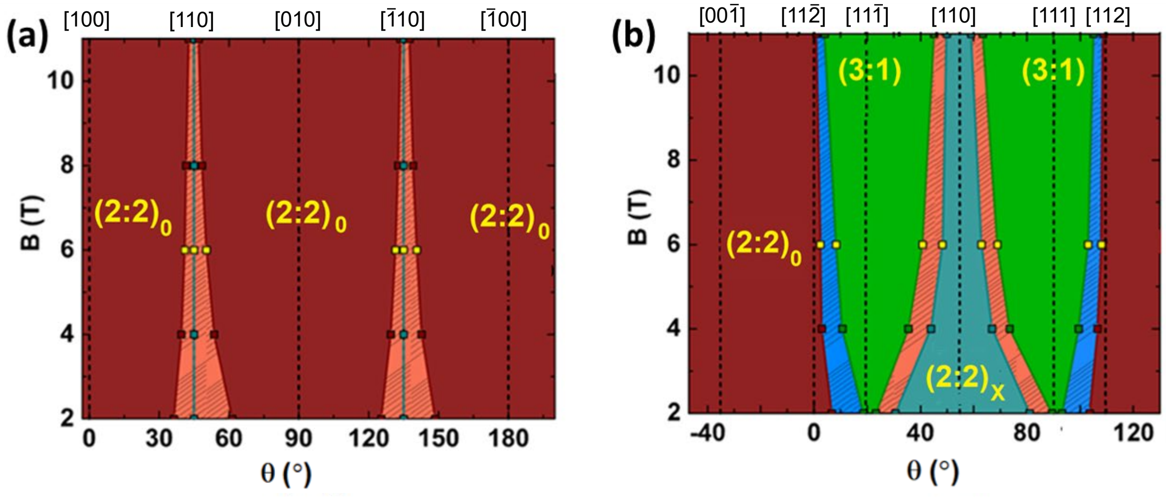

Figure 2. Angular-Field phase diagram, for applied fields ranging from 2-11 T while **B** rotates within the (a) (001) plane and (b) the $(1\bar{1}0)$ plane. The (2:2)$_0$, (2:2)$_X$, and (3:1) phases are indicated by the wine, cyan, and green colors, respectively. The blue and orange hatched regions indicate the angular ranges of domain states that form when the system transitions between stable phases. Crystallographic directions are indicated with vertical dashed lines.

and β-spins are involved in the phase transition between the (2:2)$_X$ and (3:1) monopole phases. We define

$$\frac{[1,\bar{1},0]}{\sqrt{2}} = \hat{i}';\quad \frac{[1,1,\bar{2}]}{\sqrt{6}} = \hat{j}';\quad \frac{[1,1,1]}{\sqrt{3}} = \hat{k}' \text{ (with } \hat{i}' \times \hat{j}' = \hat{k}') \tag{2a}$$

$$\mathbf{B} = B[\cos\theta\ \hat{j}' + \sin\theta\ \hat{k}'] \tag{2b}$$

$$\tau_n = \hat{n}\cdot\tau = \frac{[\bar{1},1,0]}{\sqrt{2}}\cdot(\mathbf{m}\times\mathbf{B}) \tag{2c}$$

Table 2. Functional forms for torque curves for the magnetic spin textures associated with the $(1\bar{1}0)$ rotational plane that are shown as solid curves in Figure 3 of the main text. Note, the magnetization, **m**, is given as the magnetization per one unit cell, i.e., for 16 Ho^{3+} sites, with μ=10μ_B per site. Transitions between the indicated spin textures requires reversal of 4 α- or β-spins, as indicated in blue and red text, respectively.

Phase	**B**-direction	$\mathbf{m} = \sum_{i=1}^{16} \mathbf{m_i}$		$\tau_n = \frac{[\bar{1},1,0]}{\sqrt{2}}\cdot(\mathbf{m}\times\mathbf{B})$
(2:2)$_0$ phase	$[00\bar{1}]$	$\frac{\mu}{\sqrt{3}}(0,0,-16)$		$\frac{\mu B}{6}(-32\cos\theta - 32\sqrt{2}\sin\theta)$
(3:1) monopole phase	$[11\bar{1}]$	$\frac{\mu}{\sqrt{3}}(8,8,-8)$	4xα	$\frac{\mu B}{6}(16\cos\theta - 32\sqrt{2}\sin\theta)$
(2:2)$_X$ phase	$[110]$	$\frac{\mu}{\sqrt{3}}(8,8,0)$	4xβ	$\frac{\mu B}{6}(32\cos\theta - 16\sqrt{2}\sin\theta)$
(3:1) monopole phase	$[111]$	$\frac{\mu}{\sqrt{3}}(8,8,8)$	4xβ	$\frac{\mu B}{6}(48\cos\theta)$
(2:2)$_0$ phase	$[001]$	$\frac{\mu}{\sqrt{3}}(0,0,16)$	4xα	$\frac{\mu B}{6}(32\cos\theta + 32\sqrt{2}\sin\theta)$

Supplementary Table 2 lists all the moment vectors and the functional forms of the angular dependence of the torque curves for the stable spin textures. When an α-spin flips, each flip leads to ΔS_x=ΔS_y = $\pm$2 and ΔS_z = $\pm$2. When a β-spin flips, each flip

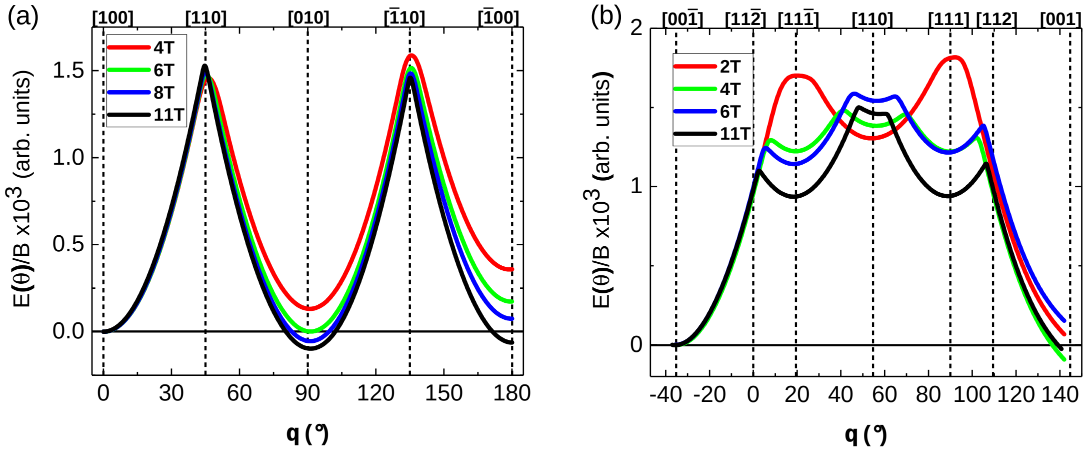

Figure 3. Field scaled integrated torque curves based on the curves shown in Figure 3 of the main manuscript. These curves represent contours on the magnetic anisotropy energy surface as a function of angle. The sample is rotated so that the field stays in (a) the (001) plane and (b) in the $(1\bar{1}0)$ plane. Crystallographic directions are indicated with vertical dashed lines.

leads to $\Delta S_z = \pm 2$ while S_x and S_y remain unchanged. After every α- or β-spin flip in a unit cell consisting of four tetrahedra, the system goes into a mixed domain phase where 25% of the system has made the phase transition (i.e., one tetrahedron in every unit cell has transitioned between a Pauling and a monopole state). Therefore, the Pauling:monopole volume fraction changes from 4:0 $\rightarrow$ 3:1 $\rightarrow$ 2:2 $\rightarrow$ 1:3 $\rightarrow$ 0:4 during a phase transition. This provides the appropriate scaling factors to extract the magnetic moment **m**, and hence, the functional form of the torque equations for the intermediate phases during the phase transition. The torque curves for the ordered phases and these intermediate spin textures are shown in Figure 3 in the main text as solid and dotted curves, respectively. The field-angle phase diagram for the $(1\bar{1}0)$ rotational plane as determined from the CTM measurements, is provided in Supplementary Figure 2(b).

3 Integrated torque curve and the magnetic anisotropy energy

From the measured torque response we can extract the anisotropy energy (E_a) as a function of field strength and direction. The magnetic torque is the derivative of this energy,

$$\tau = -\frac{dE_a}{d\theta} \tag{3}$$

where θ is the rotation angle around a defined axis within the crystal. Measuring torque responses for different rotation planes thus provide insight into the shape of the anisotropy energy surface. In most systems this torque provides a direct means to determine the magneto-crystalline anisotropy, as most magnetic materials have a rather small crystal field that can be easily overcome by an applied field. Since, the spin-ice system has a very large crystal field splitting[2], rather than measuring the magneto-crystalline anisotropy surface, we are probing the energy surface associated with the various noncollinear spin textures that are stabilized under different magnetic field conditions. Integrating the torque curves reveal the relative energy differences between these possible spin textures as the sample is rotated in applied fields. Field-scaled integrated torque curves for both the (001) and $(1\bar{1}0)$ planes are plotted in Supplementary Figure 3 for various applied field strengths.

We assume that the energy surface is continuous. Thus, it becomes clear that the $(2{:}2)_X$ phase, which is expected to form when the field is applied along any of the $\langle 110\rangle$ directions, resides on a sharp energy maximum (i.e., is not stable) when rotating in the (001) plane, yet it resides on a local minimum in the energy surface when rotating in the $(1\bar{1}0)$ plane. It appears to reside on a saddle point in energy. Based on this energy surface, a long-range ordered $(2{:}2)_X$ phase does not appear to form when the field is confined to rotate in the (001) plane, yet an ordered $(2{:}2)_X$ phase does form when the field is confined to rotate in the $(1\bar{1}0)$ plane, with the angular range of this phase clearly showing a strong dependence on field-strength.

4 The field sweep: phase transition between (3:1) and $(2{:}2)_X$

For the field sweep measurement, a similar procedure is used to calculate the torque curves as a function of field strength. For these measurements, the field is misaligned away from the $[111]$ direction by a small angle of $\Delta\theta \approx 5^\circ$ towards the $[110]$

direction. We express the applied field as $\mathbf{B} = B[\cos(\theta)\ \hat{j}' + \sin(\theta)\ \hat{k}']$ and $\tau_n = \hat{n}\cdot\tau = \frac{[\bar{1},1,0]}{\sqrt{2}}\cdot(\mathbf{m}\times\mathbf{B})$, identical to the reference frame used for the $(1\,\bar{1}\,0)$ rotation. We note that θ, defined as the angle between $\mathbf{B}$ and the $[1\,1\,\bar{2}]$ direction, is fixed during the measurement (θ = 90° - $\Delta\theta$), while B varies between 7 T and -7 T. Supplementary Table 3 lists all the moment vectors and the torque curves for the stable spin textures. It also indicates which and how many spins per unit cell are flipped during a phase transition. When a β-spin flips, each flip leads to $\Delta S_z = \pm 2$, while S_x and S_y remain unchanged whereas, an α-spin flip leads to ΔS_x=$\Delta S_y = \pm 2$ with S_z remaining unchanged.

Assuming $\Delta\theta = 5°$, the experimentally measured ratio of the slopes between the (3:1) phase and the $(2{:}2)_X$ phases gives a value of $\frac{|\mathbf{m}|_{sat}^{(3:1)}}{|\mathbf{m}|_{sat}^{(2:2)X}} \approx 1.18$. This is extremely close to the expected value of $\frac{|\mathbf{m}|_{sat}^{(3:1)}}{|\mathbf{m}|_{sat}^{(2:2)X}} = \frac{5.0\mu_B}{4.1\mu_B} = 1.22$ based on magnetization measurements reported by others[3,4]. An agreement within 5 % between the expected and experimental values gives us a great deal of confidence in the measurement set-up and phenomenological model. We also recognize that there is no stable phase when the field reverses its orientation. This occurs between -0.1 T → -1.8 T and 0.1 T → 1.8 T when all α-spins flip. However, approaching the same region from the high field end (±7 T) results into a clean $(2{:}2)_X$ phase, which follows after the β-spins flip. Because different sets of spin-flips are involved during the low and high field transitions in the field sweep measurement, a hysteretic butterfly loop within the field range of -1.8 T< B <1.8 T results. The energy cost for a spin flip to occur and create the (3:1) state on a single tetrahedron is likely too high at low field, i.e., when the (3:1) state is inaccessible, hysteresis appears, with the 8-α spins likely flipping in pairs.

Table 3. Functional forms for torque curves for the magnetic spin textures associated with the field sweep measurements. For the solid curves in Figure 4(b) of the main text, with θ = 85°. The magnetization, $\mathbf{m}$, is given as the magnetization per one unit cell, i.e., for 16 Ho^{3+} sites, with μ=10μ_B per site. Transitions between the indicated spin textures requires reversal of 8 α- or 4 β-spins, as indicated in blue and red text, respectively.

Field range (sweep direction)	Phase	$\mathbf{m} = \sum_{i=1}^{16} \mathbf{m_i}$		$\tau_n = \frac{[\bar{1},1,0]}{\sqrt{2}}\cdot(\mathbf{m}\times\mathbf{B})$
3 T< B < 7 T (Down)	(3:1) monopole	$\frac{\mu}{\sqrt{3}}(8,8,8)$		$\frac{\mu B}{6}(48\cos\theta)$
-0.1 T< B < 1.8 T (Down)	$(2{:}2)_X$	$\frac{\mu}{\sqrt{3}}(8,8,0)$	4xβ	$\frac{\mu B}{6}(32\cos\theta - 16\sqrt{2}\sin\theta)$
-1.8 T< B < -0.1 T (Down)	No stable phase	$\frac{\mu}{\sqrt{3}}(-8,-8,0)$	8xα	No stable phase
-7 T< B < -3 T (Down/Up)	(3:1) monopole	$\frac{\mu}{\sqrt{3}}(-8,-8,-8)$	4xβ	$\frac{\mu B}{6}(-48\cos\theta)$
-1.8 T< B < 0.1 T (Up)	$(2{:}2)_X$	$\frac{\mu}{\sqrt{3}}(-8,-8,0)$	4xβ	$\frac{\mu B}{6}(-32\cos\theta + 16\sqrt{2}\sin\theta)$
0.1 T< B < 1.8 T (Up)	No stable phase	$\frac{\mu}{\sqrt{3}}(8,8,0)$	8xα	No stable phase
3 T< B < 7 T (Up)	(3:1) monopole	$\frac{\mu}{\sqrt{3}}(8,8,8)$	4xβ	$\frac{\mu B}{6}(48\cos\theta)$

5 Temperature dependent hysteresis in torque magnetometry

5.1 The $(0\,0\,1)$ rotational plane

As shown in Figure 3 (a) in the main text, the steep transition around the $\langle 1\,1\,0\rangle$ family of directions develops obvious hysteresis at low applied fields at T = 500 mK. The hysteresis is a clear sign of the glassiness of the system. The field rotation speed may be important in determining the degree of hysteresis, as the system will be effectively out of equilibrium if the field rotates too fast. Here we note that hysteresis is also present at higher fields, but gradually closes with increasing field. We have also investigated how the hysteresis changes with increasing temperature (for B = 2 T), Supplementary Figure 4(a) shows the torque response at both T = 500 mK as well as T = 1.6 K. The wine and blue solid lines correspond to calculated torque curves associated with the $(2{:}2)_0$ and $(2{:}2)_X$ phases, respectively (see Supplementary Table 1). It is clear that the hysteresis disappears with increasing temperature. Therefore, we associate the appearance of the hysteresis with the local internal fields (spin-spin interactions) of the spin-ice state as it appears below the spin freezing temperature.

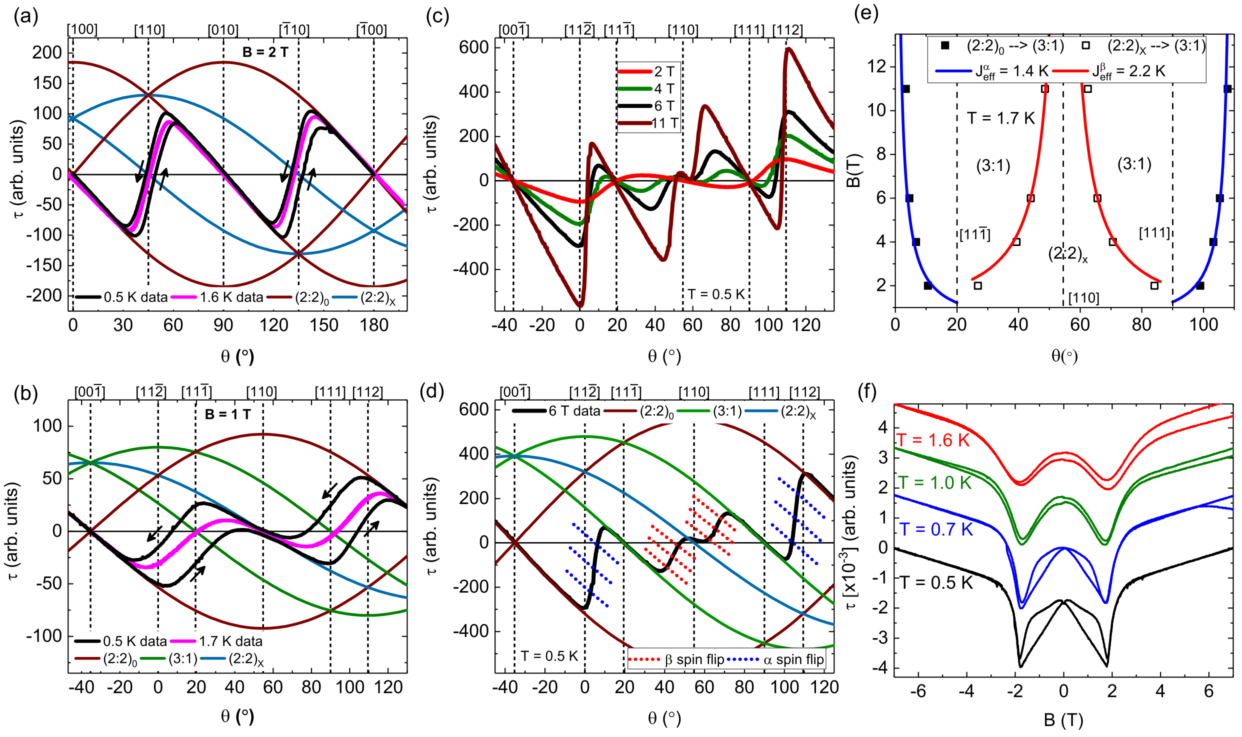

Figure 4. (a) The (001) rotational plane: CTM measurement showing torque as a function of angle at B = 2 T, measured at T = 0.5 K (solid black curve) and T = 1.6 K (solid magenta curve). The arrows indicate the sweep directions. The wine and blue solid lines are calculated torque curves associated with the (2:2)$_0$ and (2:2)$_X$ phases, respectively (see Supplementary Table 1). (b) The $(1\bar{1}0)$ rotational plane: CTM measurement showing torque as a function of angle at B = 1 T, measured at T = 0.5 K (solid black curve) and T = 1.7 K (solid magenta curve). The arrows indicate the sweep directions. The wine, blue and green solid lines are calculated torque curves associated with the (2:2)$_0$, (2:2)$_X$, and (3:1) monopole phases, respectively (see Supplementary Table 2). (c) CTM response at T = 1.7 K when **B** rotates in the plane containing the body diagonal (the $[11\bar{2}]$ direction corresponds to 0°). (d) CTM response in a 6 T applied field for the $(1\bar{1}0)$ plane at T = 1.7 K. The wine, blue and green solid lines are calculated torque curves associated with the (2:2)$_0$, (2:2)$_X$, and (3:1) monopole phases, respectively (see Supplementary Table 2). (e) Critical angles as a function of applied field for the $(1\bar{1}0)$ plane rotation data at 1.7 K for transitions between (2:2)$_0$ ⇔ (3:1) (solid squares) as well as (2:2)$_X$ ⇔ (3:1) (open squares). The blue and red curves correspond to model curves using J^{α}_{eff} = 1.4 K and J^{β}_{eff} = 2.2 K, respectively. Crystallographic directions are indicated by vertical dashed lines. (f) Capacitance change as a function of applied field, misaligned away from the $[111]$ direction at T = 0.5 K (black), T = 0.7 K (blue), T = 1.0 K (green), and T = 1.6 K (red). The curves are vertically translated for clarity.

5.2 The $(1\bar{1}0)$ rotational plane

As mentioned in the main text and as shown in Supplementary Figure 4(b), the transitions around the $[112]$ and $[11\bar{2}]$ directions between the (2:2)$_0$ and (3:1) monopole phase disappear below B = 2 T, i.e., the (3:1) monopole phase does not form at low field. As a result hysteresis appears around the $[111]$ and $[11\bar{1}]$ directions for rotations in B = 1 T. This hysteresis is clearly associated with the spin-ice phase as it has fully disappeared at T = 1.7 K (the solid magenta curve in Supplementary Figure 4(b)). The wine, blue, and green solid lines correspond to calculated torque curves associated with the (2:2)$_0$, (2:2)$_X$ and (3:1) monopole phases, respectively (see Supplementary Table 2). We associate the appearance of the hysteresis with the local internal fields (spin-spin interactions) of the spin-ice state and assume that in this rotational plane it also appears below the spin freezing temperature.

In Supplementary Figure 4(c) we show the CTM response measured at 1.7 K in various applied fields. Using the same procedure as described in the main text we compare these angular sweeps with the calculated torque curves and extract the critical angles for the (2:2)$_0$ ⇔ (3:1) and (2:2)$_X$ ⇔ (3:1) transitions (see Supplementary Figure 4(d)). The critical angles as a function of applied field are plotted in Supplementary Figure 4(e). The thermal smearing of the transitions and the fact that at 2 T we do not observe the (3:1) state, makes the analysis as described in the main text difficult for the high temperature data.

Instead of fitting the critical angles, we plot them together with the functional forms for $B(\theta)$ using 1.4 and 2.2 K for J^{α}_{eff} and J^{β}_{eff}, respectively. The agreement between $B(\theta)$ and the data is very good.

5.3 Temperature dependent hysteresis in field sweep with $\mathbf{B}$ near the $[1\,1\,1]$ direction

We have also investigated how the field sweep changes as the temperature is gradually increased from T = 500 mK to T = 1.6 K (see Supplementary Figure 4(f)). For these measurements, the field is misaligned away from the $[1\,1\,1]$ direction by a small angle of $\Delta\theta \approx 5^\circ$ towards the $[1\,1\,0]$ direction. While the linear responses at high fields ($B > 2$ T) and at low fields (0 T $< B <$ 1.5 T) remain, the sharp turnover around B = 1.8 T gradually smears out at higher temperature. The hysteresis around zero field disappears fully when the sample is heated to above T = 0.7 K, i.e., around the spin freezing temperature. We observe no hysteresis around the sharp feature at B = 1.8 T, which is expected and consistent with reports by others[3]. We also notice that as the temperature is increased, the value for ΔC at the turnover (B = 1.8 T) decreases in amplitude. This is consistent with the formation of thermal defects in the $(2{:}2)_X$ phase (with $\mathbf{S} = (8,8,0)$) resulting in a gradual increase in S_z as the long-range antiferromagnetic order on the β-spins gets randomized. Additionally, there is a drift in the measurement at 1.6 K (a little drift is observed at 1 K as well) and we associate this trend with a systematic drift in the sample temperature during field sweep. So, there is a clear evidence that the capacitance picks up an additional term because of the temperature variation during the field sweep sequence. This is expected in a sorption-pump based He3 cooling system because higher field burns helium at a faster rate.

6 Hamiltonian and Monte Carlo Simulations

The Hamiltonian of interest for $Ho_2Ti_2O_7$ is,

$$H = -J_1 \sum_{\langle i,j\rangle} \tilde{\mathbf{S}}_i \cdot \tilde{\mathbf{S}}_j - J_2 \sum_{\langle\langle i,j\rangle\rangle} \tilde{\mathbf{S}}_i \cdot \tilde{\mathbf{S}}_j + D r_{nn}^3 \sum_{i>j} \left(\frac{\tilde{\mathbf{S}}_i \cdot \tilde{\mathbf{S}}_j}{|\mathbf{r}_{ij}|^3} - \frac{3(\tilde{\mathbf{S}}_i \cdot \mathbf{r}_{ij})(\tilde{\mathbf{S}}_j \cdot \mathbf{r}_{ij})}{|\mathbf{r}_{ij}|^5} \right) - g\mu_B \sum_i \mathbf{B} \cdot \tilde{\mathbf{S}}_i \tag{4}$$

where $\tilde{\mathbf{S}}_i$ are classical spin vectors with $|\tilde{\mathbf{S}}_i| = 1$. The tilde is used to indicate that the spins are constrained to point along the local $\langle 1\,1\,1\rangle$ axis of the tetrahedra they belong to. $\mathbf{r}_i$ is the real-space location of site i, $\mathbf{r}_{ij} \equiv \mathbf{r}_i - \mathbf{r}_j$, $\langle i,j\rangle$ ($\langle\langle i,j\rangle\rangle$) refers to nearest-neighbor (next nearest-neighbor) bonds, r_{nn} is the nearest-neighbor bond distance, J_1 (J_2) is the nearest-neighbor (next-nearest neighbor) interaction strength, and D is the strength of the long-range dipolar term. The $i > j$ notation guarantees each of pair of spins is only counted once. $g\mu_B$ is the size of the magnetic moment and $\mathbf{B}$ is the applied magnetic field.

The spins are effectively Ising-like since they are constrained to be aligned along a local $\langle 1\,1\,1\rangle$ axis, i.e.,

$$\tilde{\mathbf{S}}_i = \sigma_i z^{a(i)} \tag{5}$$

where $\sigma_i = \pm 1$ is a scalar and $z^{a(i)}$ is a unit vector depending on the sublattice $a = 0,1,2,3$ to which the site i belongs. The directions of the z^a vectors are shown in Supplementary Table 4.

Table 4. Sublattice unit vectors

a (sublattice)	$z^a = (x,y,z)$
0	$\frac{1}{\sqrt{3}}(+1,+1,+1)$
1	$\frac{1}{\sqrt{3}}(+1,-1,-1)$
2	$\frac{1}{\sqrt{3}}(-1,+1,-1)$
3	$\frac{1}{\sqrt{3}}(-1,-1,+1)$

When the dipolar interaction is truncated to the nearest neighbor term, the Hamiltonian becomes,

$$H_{NN} = J^{s-DSI}_{1,eff} \sum_{\langle i,j\rangle} \sigma_i \sigma_j - g\mu_B \sum_i \sigma_i \mathbf{B} \cdot z^{a(i)} \tag{6}$$

where $J^{s-DSI}_{1,eff} \equiv \left(\frac{J_1+5D}{3}\right)$. For the case of zero applied field, $J^{s-DSI}_{1,eff} > 0$ results in the "2 in-2 out ice rule" for the ground state manifold of the spin ice.

We perform classical Monte Carlo (MC) simulations for both the nearest-neighbor (NN) and the standard dipolar spin-ice (s-DSI) models, with the parameters shown in Supplementary Table 5. The s-DSI model includes long-range dipolar interactions via the Ewald summation method[5–8], the parameter set for the s-DSI model is taken from Refs.[9,10]

Table 5. The Parameter values for J_1, D, and g used in the MC simulations for the NN and s-DSI model, respectively. The values for $J_{1,eff}^{s-DSI}$ are provided as a reference, but they are not used in the simulations.

Model	J_1 (K)	D (K)	$J_{1,eff} = \frac{J_1+5D}{3}$ (K)	g (dimensionless)
NN	+5.40	0	1.80	10
s-DSI (NN dipolar)	-1.56	1.41	1.83	10

Table 6. The table summarizes values for J_1, J_2, J_3, D, and $J_{1,eff}^{s-DSI}$ as reported by others[11–14]. J_{3a} and J_{3b} refer to third nearest neighbors of a and b types, respectively (see refs.[11, 15]).

Ref	J_1 (K)	J_2 (K)	J_{3a} (J_{3b}) (K)	D (K)	$J_{1,eff} = \frac{J_1+5D}{3}$ (K)
DTO[11]	3.41	-0.14	0.025 (0.025)	1.32	1.06
DTO[12]	3.41	0	0.014 (0.096)	1.32	1.06
DTO[13]	-3.72	-	-0.02	1.41	1.11
HTO[13]	-1.65	-	-	1.41	1.80
HTO[14]	-1.56	-	-	1.41	1.83

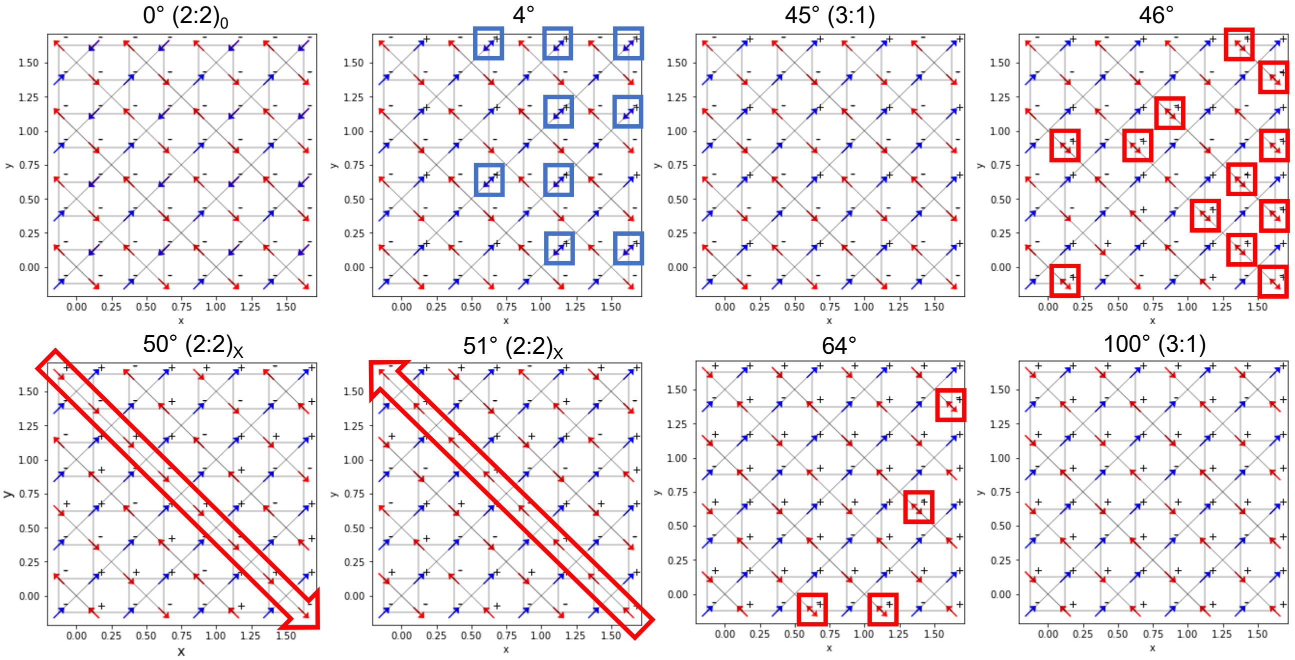

Figure 5. Snapshots of the spin configurations recorded for various angles between the applied field and the [0 0 1] direction. The snapshots are 2-dimensional projections of the 2x2x2 unit cells, projected down the z-axis (β-spins are red and the α-spins are blue). The stable phases are indicated, $(2{:}2)_0$ at 0°(when **B** is along the [001]), (3:1) at 45°and 100°, and $(2{:}2)_X$ at 50°and 51°. The open red arrows highlight the flipped directions of the β-spin chains. At the intermediate angles of 4°, 46°, and 64°, the spin textures adopt an intermediate state in which the two stable phases involved in the transition form a domain structure. The blue and red boxes highlight the spin columns that host unflipped α- and β-spins, respectively.

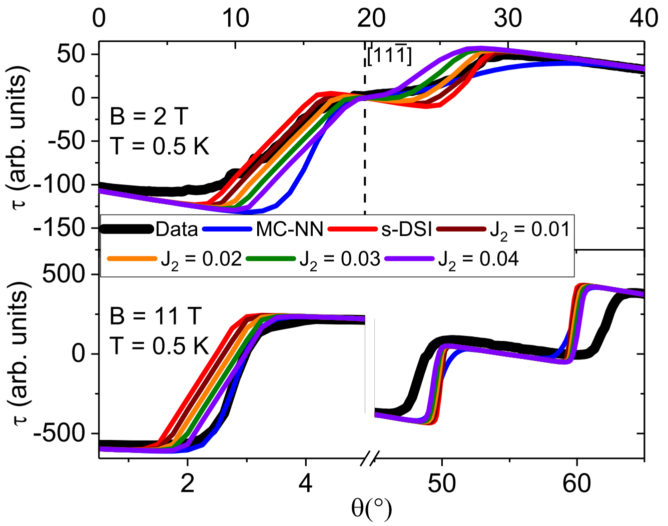

Figure 6. Simulated torque response as a function of angle, obtained from MC simulations using the nearest neighbor model (NN, blue curves), s-DSI model (red curves) and the generalized DSI model (s-DSI with J_2 = 0.01 meV, 0.02 meV, 0.03 meV, and 0.04 meV, wine, orange, green, and purple curves, respectively); measured CTM data (black curves) are taken at T = 0.5 K in applied fields of 2 T (top panel) and 11 T (bottom panel).

In Supplementary Table 6 we compare our values to others reported for HTO and $Dy_2Ti_2O_7$ (DTO).

We simulate finite size pyrochlore clusters with periodic boundary conditions with $N_{spins} = 16 \times 4^3 = 1024$ lattice sites and employ the Ewald summation method[5–8]. Demagnetization effects[6] are found to be significant especially for field directions close to the observed phase boundaries. We model this with the help of a surface term in the Ewald summation[7], $U^{surf} = Dr_{nn}^3 \frac{2\pi}{(2\mu'+1)N_{spins}} \sum_{i,j} \tilde{\mathbf{S}}_i \cdot \tilde{\mathbf{S}}_j$. Incorporating this term with $\mu' = 1$ provides a good match to the experimental observations.

Our MC sampling procedure employs the standard accept-reject Metropolis algorithm with an equal probability of “loop” moves (effective for sampling the low energy spin-ice configurations at low temperature) [6,16,17], and single spin flip moves (effective at high temperature). We measure the thermal average of the magnetization $\langle \mathbf{m} \rangle$ (per unit cell) using 10^5 MC samples. One sample per MC sweep is recorded for thermal averaging, and one MC sweep comprises of a set of N_{spins} proposed moves (which are individually of loop or single spin flip type). We then compute the component of the torque along $\hat{n}$ (per unit cell) $\tau_n \equiv \hat{n} \cdot (\langle \mathbf{m} \rangle \times \mathbf{B})$, as discussed in the previous section. This quantity corresponds directly to what is being measured in the torque magnetometry experiment (up to device specific constants). We scan the space of field alignment angles in steps of 1°(or smaller near phase boundaries), considering the same range of angles as that recorded in the experiment.

In Supplementary Figure 5 we show snapshots of spin textures recorded during the MC simulations at T = 0.5 K as the field was rotated from the [001] direction at 0°. We show snapshots at various relevant angles, observing the expected stable phases $(2{:}2)_0$ at 0°, (3:1) at 45°and 100°, and $(2{:}2)_X$ at 50°and 51°. The reason that two $(2{:}2)_X$ configurations are shown is because in the 47 - 63°range, the spin texture toggles between the two possible states with oppositely oriented β-spin chains every couple of degrees, indicating that these textures are indeed degenerate. No defects are observed in either of the $(2{:}2)_X$ configurations, suggesting that thermal fluctuations at T = 0.5 K are small and the state is relatively stable. Lastly, the configurations taken at angles of 4°, 46°, and 64°show intermediate or transient spin textures in which the two stable phases involved in the transition form a domain structure. This is visible from the presence of the double arrows, which indicates that in that column of spins, both in and out spins (either unflipped α- or β- spins) exist.

In Supplementary Figure 6, simulated torque curves are compared to the experimental curves taken at 0.5 K in 2 and 11 T, respectively. The results for a strictly nearest neighbor model (NN, blue curve) and for the s-DSI (long-range dipolar) model (including Ewald summation, loop moves, and demagnetization effects[6], red curve) are shown. The wine, orange, green, and purple curves were simulated with a more generalized DSI model using s-DSI model parameters and including various values for the J_2 interaction (0.01, 0.02, 0.03, and 0.04 meV).

7 Calculation of total energies of the spin-ice states and estimates of $J_{eff}^{\alpha/\beta}$

We have calculated the total energy of each of the spin-ice states that we observed in the CTM experiment and the MC simulations. in the framework of a short-range model that includes $J_{1,eff}$, $J_{2,eff}$, $J_{3a,eff}$ and $J_{3b,eff}$. The zero field part of this

effective Hamiltonian is,

$$H = J_{1,eff} \sum_{\langle i,j \rangle} \sigma_i \sigma_j + J_{2,eff} \sum_{\langle\langle i,j \rangle\rangle} \sigma_i \sigma_j + J_{3a,eff} \sum_{\langle\langle\langle i,j \rangle\rangle\rangle,a} \sigma_i \sigma_j + J_{3b,eff} \sum_{\langle\langle\langle i,j \rangle\rangle\rangle,b} \sigma_i \sigma_j \tag{7}$$

where $\langle\langle\langle i,j \rangle\rangle\rangle, a$ and $\langle\langle\langle i,j \rangle\rangle\rangle, b$ refer to third nearest neighbors of a and b types respectively. (The a type third nearest neighbors share a common neighbor and are thus connected to each other via a minimum of two "hops" on the pyrochlore lattice. The b type third nearest neighbors do not share a nearest neighbor, they are instead related by a path involving a minimum of three hops on the pyrochlore lattice[11,15].)

We enumerated the first, second and third nearest neighbors (of both kinds a, b) for the pyrochlore lattice. (This was done numerically for a cubic unit cell of $16 \times 2^3 = 128$ sites). We computed the Zeeman energy and the interaction energy per 16-site unit cell for three phases: (2:2)$_0$ (with $\mathbf{m}||z$), (2:2)$_X$, and 3:1 ($+---$, i.e., 3-in 1-out).

The sum of the interaction and Zeeman contributions (i.e., the second term in Supplementary Equation 6 with $\mathbf{B}$ defined as (h_x,h_y,h_z)) gives us the total energy (which per 16-site cubic unit cell) is as below:

$$(2:2)_0 : -16J_{1,eff} - 32J_{2,eff} + 48J_{3a,eff} + 48J_{3b,eff} - \frac{16}{\sqrt{3}} g\mu_B h_z \tag{8a}$$

$$(2:2)_X : -16J_{1,eff} + 16J_{3a,eff} + 16J_{3b,eff} - \frac{8}{\sqrt{3}} g\mu_B (h_x + h_y) \tag{8b}$$

$$(3:1) : +48J_{3a,eff} + 48J_{3b,eff} - \frac{8}{\sqrt{3}} g\mu_B (h_x + h_y + h_z) \tag{8c}$$

The magnetic field components as a function of the angle (θ) are (see Supplementary Equations 2(a) and (b)):

$$h_x = \Big(\frac{\cos\theta}{\sqrt{6}} + \frac{\sin\theta}{\sqrt{3}}\Big) B \tag{9a}$$

$$h_y = \Big(\frac{\cos\theta}{\sqrt{6}} + \frac{\sin\theta}{\sqrt{3}}\Big) B \tag{9b}$$

$$h_z = \Big(-\frac{2\cos\theta}{\sqrt{6}} + \frac{\sin\theta}{\sqrt{3}}\Big) B \tag{9c}$$

To obtain the phase boundary of the $(2:2)_0 \Leftrightarrow (3:1)$ transition at zero temperature ($T = 0$) we equate the total energies of the two phases (per 16-site unit cell). We get,

$$2J_{1,eff} + 4J_{2,eff} = \frac{1}{\sqrt{3}} g\mu_B (h_x + h_y - h_z) \tag{10}$$

The phase transitions between symmetry related phases (2:2)$_0$ (with $\mathbf{m}||-z$) and 3:1 ($+++-$, i.e., 3-out 1-in) can be evaluated in a similar way. Note that the $J^a_{3,eff}$ and $J^b_{3,eff}$ terms cancel out for this transition. Thus, J_3 interactions do not affect this transition.

The effective parameters can be expressed in terms of the original generalized DSI model (now incorporating second and third nearest neighbors with couplings J_2, J_{3a}, and J_{3b}) following Ref.[15], who considered the case of $J_2 = J_{3a} = J_{3b} = 0$, but with the opposite sign convention of J_1 compared to our work.The relations are,

$$J_{1,eff} \equiv \frac{J_1 + 5D}{3} \tag{11a}$$

$$J_{2,eff} \equiv \frac{J_2 - D/\sqrt{3}}{3} \tag{11b}$$

$$J_{3a,eff} \equiv -J_{3a} - \frac{D}{8} \tag{11c}$$

$$J_{3b,eff} \equiv -J_{3b} + \frac{D}{8} \tag{11d}$$

The factor of $1/3$ arises from the geometric factor $z_i \cdot z_j = -1/3$ for two spins on different sublattices as is the case for nearest and second nearest neighbors. The third nearest neighbors are on the same sublattice for which $z_i \cdot z_i = 1$. Note that this model truncates the dipolar interaction, and is thus expected to be only qualitatively accurate. Based on Supplementary Equations 11 and 10 we can extract a J^{α}_{eff} that would correspond to this combination of interaction terms.

$$J^{\alpha}_{eff} \equiv J_{1,eff} + 2J_{2,eff} = \frac{1}{3}\Big(J_1 + 2J_2 + \Big(5 - \frac{2}{\sqrt{3}}\Big)D\Big) \tag{12}$$

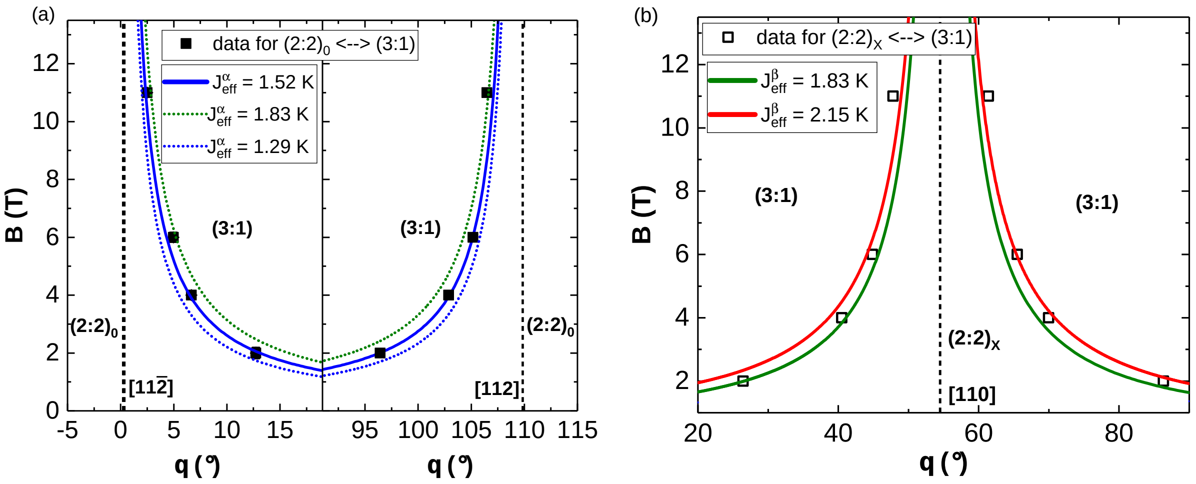

Figure 7. (a) Critical angles extracted from CTM data as a function of applied field for the $(1\,\bar{1}\,0)$ plane rotation for the (2:2)$_0$ ⇔ (3:1) transitions (solid squares). This is the same data as was plotted in the main text Figure 4(a). Calculated phase boundaries for this transition using Supplementary Equations 10 and 12, the solid blue line represents the model using J^{α}_{eff} = 1.52 K (with J_1=-1.56 K, J_2 = 0.35 K, and D=1.41 K); the green dotted line represents the model for which $J_{2,eff}$=0 and $J_{1,eff}$=1.83 K (with J_1=-1.56 K and D=1.41 K (the NN-model)); the blue dotted line represents the model using Supplementary Equation 12 but (with J_1=-1.56 K, J_2=0, and D=1.41 K, i.e., dipolar interactions are truncated at the second nearest neighbor). (b) Critical angles extracted from CTM data as a function of applied field for the $(1\,\bar{1}\,0)$ plane rotation for the (2:2)$_X$ ⇔ (3:1) transitions (open squares). This is the same data as was plotted in the main text Figure 4(a). Calculated phase boundaries for this transition using Supplementary Equations 13 and 14. The solid green line represents the model using J^{β}_{eff} = 1.83 K with J_1=-1.56 K, (J_{3a}+J_{3b})=0, and D=1.41 K (the NN-model). The red solid line represents the model using Supplementary Equation 14 with J_1=-1.56 K, D=1.41 K, and (J_{3a}+J_{3b})=-0.16 K.

For $J_1 = -0.13499$ meV (-1.56 K), $D = 0.121567$ meV (1.41 K), and $J_2 = 0.030$ meV (0.35 K) we get J^{α}_{eff} = 1.52 K, which is lower than $J_{1,eff}$=1.83 K, encouragingly it is in the same ballpark as that measured in the CTM experiment. Hence, once the interaction terms are known, we can rearrange Supplementary Equation 10 and plot B as a function of θ, which represents the phase boundary at which the transition occurs (see Supplementary Figure 7 (a)). We plot the phase boundary for three combinations of J_1, J_2, and D.

We repeat the analysis for the (2:2)$_X$ ⇔ (3:1) transition. On equating their energies, we get

$$2J_{1,eff} + 4J_{3a,eff} + 4J_{3b,eff} = \frac{1}{\sqrt{3}} g\mu_B h_z \tag{13}$$

Based on Supplementary Equations 13 and 11, we can extract a J^{β}_{eff} that would correspond to this combination of interaction terms.

$$J^{\beta}_{eff} \equiv J_{1,eff} + 2(J_{3a,eff} + J_{3b,eff}) = \frac{J_1 + 5D}{3} - 2(J_{3a} + J_{3b}) \tag{14}$$

We can thus use the field dependent critical angles obtained by CTM to constrain $J_{3a} + J_{3b} \approx -0.014$ meV (-0.16 K) by solving Supplementary Equation 13 for multiple field strengths and looking at the value that best agrees with the experimental data. This value of $J_{3a} + J_{3b}$ corresponds to a J^{β}_{eff} of about 2.15 K. Note however that this argument alone does not constrain J_{3a} and J_{3b} individually. In Supplementary Figure7 (b), the calculated phase boundaries are shown for the (2:2)$_X$ ⇔(3:1) transition plotted along side the critical angles extracted for the CTM data.

8 Single crystal X-ray diffraction

Single crystal X-ray diffraction was used to fully characterize the structure of the HTO single crystals. A small specimen was cut directly from a larger piece used for torque measurements and mounted to the end of a glass fiber using clear adhesive.

A full quadrant of X-ray diffraction data were collected under ambient conditions using ω-scans with 1°frame widths to a resolution of 0.4 Å, equivalent to $2\theta \sim 125$ °, on an Oxford-Diffraction Xcalibur-2 CCD diffractometer equipped with a graphite-monochromated MoK_α source. Reflections were recorded, indexed and corrected for absorption using the Oxford-Diffraction CrysAlisPro software[18], initial structure determination was performed using SHELXS[19] and final cycles of structure refinement were carried out in CRYSTALS[20]. Model refinement was performed against F^2 (i.e., structure factor intensities), and high-quality data allowed unconstrained refinement of all atom positions and anisotropic displacement ellipsoids.

Final structural refinement results and structure factors have been deposited with the joint CCDC/FIZ Karlsruhe online deposition service; these files are available for download under deposition nr. CSD-2172269[21].

Supplementary References

1. Harris, M. J., Bramwell, S. T., McMorrow, D. F., Zeiske, T. & Godfrey, K. W. Geometrical frustration in the ferromagnetic pyrochlore $Ho_2Ti_2O_7$. *Phys. Rev. Lett.* **79**, 2554–2557 (1997).
2. Opherden, L. *et al.* Magnetization beyond the ising limit of $Ho_2Ti_2O_7$. *Phys. Rev. B* **99**, DOI: 10.1103/physrevb.99.085132 (2019).
3. Krey, C. *et al.* First order metamagnetic transition in $Ho_2Ti_2O_7$ observed by vibrating coil magnetometry at milli-kelvin temperatures. *Phys. Rev. Lett.* **108**, 257204 (2012).
4. Petrenko, O. A., Lees, M. R. & Balakrishnan, G. Magnetization process in the spin-ice compound $Ho_2Ti_2O_7$. *Phys. Rev. B* **68**, 012406, DOI: 10.1103/PhysRevB.68.012406 (2003).
5. De Leeuw, S. W., Perram, J. W. & Smith, E. R. Computer simulation of the static dielectric constant of systems with permanent electric dipoles. *Annu. Rev. Phys. Chem.* **37**, 245–270, DOI: 10.1146/annurev.pc.37.100186.001333 (1986). PMID: 21819241, https://doi.org/10.1146/annurev.pc.37.100186.001333.
6. Melko, R. G. & Gingras, M. J. P. Monte carlo studies of the dipolar spin ice model. *J. Physics: Condens. Matter* **16**, R1277 (2004).
7. Wang, Z. & Holm, C. Estimate of the cutoff errors in the ewald summation for dipolar systems. *The J. Chem. Phys.* **115**, 6351–6359, DOI: 10.1063/1.1398588 (2001). https://doi.org/10.1063/1.1398588.
8. Jaubert, L. D. C. Topological Constraints and Defects in Spin Ice. PhD thesis, École Normale Supérieure de Lyon (2009).
9. Bramwell, S. T. & Harris, M. J. The history of spin ice. *J. Physics: Condens. Matter* **32**, 374010, DOI: 10.1088/1361-648x/ab8423 (2020).
10. Bramwell, S. T. & Gingras, M. Spin Ice State in Frustrated Magnetic Pyrochlore Materials. *Science* **294**, 1495, DOI: 10.1126/science.1064761 (2001).
11. Henelius, P. *et al.* Refrustration and competing orders in the prototypical $Dy_2Ti_2O_7$ spin ice material. *Phys. Rev. B* **93**, 024402, DOI: 10.1103/PhysRevB.93.024402 (2016).
12. Samarakoon, A. M. *et al.* Machine-learning-assisted insight into spin ice $Dy_2Ti_2O_7$. *Nat. Commun.* **11**, 892 (2020).
13. Ruff, J. P. C., Melko, R. G. & Gingras, M. J. P. Finite-temperature transitions in dipolar spin ice in a large magnetic field. *Phys. Rev. Lett.* **95**, 097202, DOI: 10.1103/PhysRevLett.95.097202 (2005).
14. Bramwell, S. T. *et al.* Spin correlations in $Ho_2Ti_2O_7$: A dipolar spin ice system. *Phys. Rev. Lett.* **87**, 047205 (2001).
15. Borzi, R. A. *et al.* Intermediate magnetization state and competing orders in $Dy_2Ti_2O_7$ and $Ho_2Ti_2O_7$. *Nat. Commun.* **7**, 12592, DOI: 10.1038/ncomms12592 (2016).
16. Melko, R. G., den Hertog, B. C. & Gingras, M. J. P. Long-range order at low temperatures in dipolar spin ice. *Phys. Rev. Lett.* **87**, 067203, DOI: 10.1103/PhysRevLett.87.067203 (2001).
17. Barkema, G. T. & Newman, M. E. J. Monte carlo simulation of ice models. *Phys. Rev. E* **57**, 1155–1166, DOI: 10.1103/PhysRevE.57.1155 (1998).
18. CRYSALISPRO, Rigaku Oxford Diffraction, CrysAlisPro Software system, version 1.171.38.43, Rigaku Corporation, Oxford, UK.
19. Sheldrick, G. M. A short history of SHELX. *Acta Cryst.* **A64**, 112–122 (2008).
20. Betteridge, P. W., Carruthers, J. R., Cooper, R. I., Prout, K. & Watkin, D. J. CRYSTALS version 12: software for guided crystal structure analysis. *J. Appl. Cryst.* **36**, 1487 (2003).
21. Groom, C. R., Bruno, I. J., Lightfoot, M. P. & Ward, S. C. The Cambridge Structural Database. *Acta Cryst.* **B72**, 171–179, DOI: 10.1107/S2052520616003954 (2016).
